# A Dynamic Submaximal Fatigue Protocol Alters Wrist Biomechanical Properties and Proprioception

**DOI:** 10.3389/fnhum.2022.887270

**Published:** 2022-05-30

**Authors:** Giulia A. Albanese, Valeria Falzarano, Michael W. R. Holmes, Pietro Morasso, Jacopo Zenzeri

**Affiliations:** ^1^Department of Robotics, Brain and Cognitive Sciences, Istituto Italiano di Tecnologia, Genova, Italy; ^2^Department of Informatics, Bioengineering, Robotics and Systems Engineering (DIBRIS), University of Genoa, Genoa, Italy; ^3^Faculty of Applied Health Sciences, Brock University, St. Catharines, ON, Canada

**Keywords:** wrist, biomechanics, robotic assessment, fatigue, proprioception, stiffness

## Abstract

Fatigue is a temporary condition that arises as a result of intense and/or prolonged use of muscles and can affect skilled human performance. Therefore, the quantitative analysis of these effects is a topic of crucial interest in both ergonomics and clinical settings. This study introduced a novel protocol, based on robotic techniques, to quantitatively assess the effects of fatigue on the human wrist joint. A wrist manipulandum was used for two concurrent purposes: (1) implementing the fatigue task and (2) assessing the functional changes both before and at four time points after the end of the fatigue task. Fourteen participants completed the experimental protocol, which included the fatigue task and assessment sessions over 2 days. Specifically, the assessments performed are related to the following indicators: (1) isometric forces, (2) biomechanical properties of the wrist, (3) position sense, and (4) stretch reflexes of the muscles involved. The proposed fatigue task was a short-term, submaximal and dynamic wrist flexion/extension task designed with a torque opposing wrist flexion. A novel task termination criterion was employed and based on a percentage decrease in the mean frequency of muscles measured using surface electromyography. The muscle fatigue analysis demonstrated a change in mean frequency for both the wrist flexors and extensors, however, only the isometric flexion force decreased 4 min after the end of the task. At the same time point, wrist position sense was significantly improved and stiffness was the lowest. Viscosity presented different behaviors depending on the direction evaluated. At the end of the experiment (about 12 min after the end of the fatigue task), wrist position sense recovered to pre-fatigue values, while biomechanical properties did not return to their pre-fatigue values. Due to the wide variety of fatigue tasks proposed in the literature, it has been difficult to define a complete framework that presents the dynamic of fatigue-related changes in different components associated with wrist function. This work enables us to discuss the possible causes and the mutual relationship of the changes detected after the same task.

## Introduction

According to a traditional definition proposed by the Italian physiologist Angelo Mosso (Di Giulio et al., 2006), fatigue is a disabling symptom that involves both physical and cognitive functions. The exercise-induced impairment of motor performance can be defined as performance fatigability, while the sensation of weakness that arises from prolonged muscle activity is termed perceived fatigability (Kluger et al., 2013). By defining fatigue in terms of fatigability, the level of fatigue reported by an individual depends on the rates of change in the two attributes (Enoka and Duchateau, 2008). This taxonomy is different from another dichotomy used in the field, i.e., the distinction between central and peripheral fatigue (Bigland Ritchie et al., 1978). The two components of this latter distinction are interdependent since adjustments in the activation signal discharged by motor neurons are attributable to changes occurring within the muscle. Contrarily, both performance and perceived fatigability contribute to fatigue but are due to different modulating factors. While the former is related to contractile functions and muscle activation, the latter is related to homeostasis and the psychological state of the individual (Enoka and Duchateau, 2016). Studying the effects of fatigue on human performance is a crucial research topic, which can involve a broad set of disciplines, such as ergonomics (Abdel-Malek et al., 2020), physiology (Komi and Tesch, 1979), psychology (Marcora et al., 2009), and medicine (Lou et al., 2010), with application to industrial engineering and clinical rehabilitation.

Early onset of fatigue is a common symptom of multiple sclerosis (Krupp et al., 1988; Hadjimichael et al., 2008), Parkinson’s disease (Stevens-Lapsley et al., 2012), muscle dystrophies (Angelini and Tasca, 2012), spinal cord injuries (Pelletier and Hicks, 2011), and stroke (Toffola et al., 2001), thus affecting people’s quality of life and their activities of daily living (Hadjimichael et al., 2008). In addition, fatigue can influence movement quality and injury risk in athletics and workplace tasks. Given the importance of understanding the basic mechanisms and effects of fatigue, many authors have exploited different protocols for evaluating the effects of fatigue on humans. Common protocols involve maximal or submaximal contractions, static or dynamic tasks, whose duration could be either fixed, based on the subject’s maximal level of tolerable fatigue, or based on a specified decrease in force (Kahl and Hofmann, 2016; Mugnosso et al., 2018). However, even when considering a single joint, findings related to the effects of fatigue are highly task-dependent and time-dependent (Enoka, 1995). Different tasks could lead to completely different results on the same joint, thus making it difficult to generalize about the effects of fatigue on global joint function. Considering the distal upper limb, an accurate quantitative assessment could be critical to discuss fatigue-related changes and recovery in all-around wrist function and performance. In particular, fine motor control at the wrist involves its ability to exert active muscle work, sensory skills, and biomechanical properties (Goble et al., 2012; Falzarano et al., 2021). In Kumar et al. (2020), wrist maximal voluntary isometric contraction decreased following a submaximal dynamic fatigue task, without a full recovery within 10 min after post-task termination. However, since the recovery time is dependent on both the intensity and the duration of the task (Krüger et al., 2019), it is difficult to predict how a different load or termination criterion could have influenced these results. Few and conflicting works can be found concerning the effects of fatigue on wrist biomechanical properties and position sense. Regarding biomechanical properties, it has been found that stiffness decreases and viscosity increases after an extended fatigue task (Qita and Kearney, 2000). Literature about wrist position sense shows impairments immediately after maximal dynamic exercise to exhaustion (Karagiannopoulos et al., 2020) but improvements after a submaximal dynamic task (Mugnosso et al., 2019). These studies highlight a potential dichotomy between force output, biomechanical properties and proprioception that is influenced by the parameters of a fatigue task.

In this study, we evaluated the effects of a short-term, submaximal and dynamic wrist flexion/extension fatigue task on wrist function, using a robotic device. At the end of a fatiguing robotic task involving sequences of wrist joint rotations, we evaluated the presence of fatigue-induced changes in wrist performance indicators. In particular, the testing protocol focused on assessing maximal voluntary contractions, joint biomechanical properties, and wrist position sense, before and at four precisely selected time points after the fatigue task. The primary purpose of this work was to assess how different components of wrist function are affected by forearm muscle fatigue. A secondary purpose was the development of a robust and repeatable robot-based protocol that would allow us to evaluate how different components of wrist function might be related under fatigued states. We hypothesized that maximal voluntary isometric contraction, joint biomechanical properties, and wrist position sense would demonstrate fatigue-related changes similar to those found after the previously cited submaximal dynamic fatigue protocols (Qita and Kearney, 2000; Mugnosso et al., 2019; Kumar et al., 2020). We expected improvements/impairments right after the fatigue task. However, given the intensity and duration of the task employed in this protocol, we assumed a full recovery by the last evaluation.

## Materials and Methods

### Participants

Fourteen right-handed volunteers (11 females; 27 ± 2.9 years of age) were recruited into the experimental protocol. All participants had no history of neurological disorders or previous wrist injuries. Before taking part in the study, each participant signed an informed consent. The experimental protocol was performed at the Motor Learning, Assistive, and Rehabilitation Robotics Lab of the Istituto Italiano di Tecnologia and was approved by the local ethical committee of the Liguria Region (n. 222REG2015), following the Declaration of Helsinki principles.

### Experimental Setup

The experimental setup involved the WristBot (Masia et al., 2009; Iandolo et al., 2019), a robotic manipulandum currently employed in motor control and human wrist rehabilitation studies (Albanese et al., 2021c; Mannella et al., 2021). This robotic device allows wrist movements along three degrees of freedom (DoF): flexion-extension (FE), radial-ulnar deviation (RUD), and pronation-supination (PS), with a range of motion along each DoF comparable to humans. The device is equipped with four brushless motors, activated by a control unit that allows two modes of operation: position or torque control, to provide subjects with position or torque displacements, according to the specific protocol. High-resolution incremental encoders measure the angular displacements of each DoF and a custom-made grip sensor is wrapped around the robotic handle to measure grip force (Albanese et al., 2021b; Falzarano et al., 2021). During the experimental protocol, sEMG signals were recorded from three forearm muscles using bipolar Ag/AgCl electrodes with a sampling rate of 2,048 Hz (OTBioelettronica Quattrocento). Muscles included flexor carpi radialis (FCR), flexor carpi ulnaris (FCU), and extensor carpi radialis (ECR). Kinematics, torques and sEMG signals were synchronized by a trigger signal from the robotic device to the sEMG system to relate the muscle activation to the corresponding movement.

### Experimental Protocol

**FIGURE 1:**
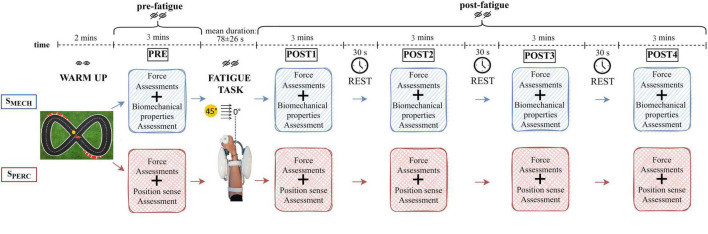
Schematic representation of the experimental protocol. Both sessions (S_*MECH*_ and S_*PERC*_) were performed by each subject on two different days in random order. Both the warm-up and the fatigue task were repeated during both sessions.

The experimental protocol included two sessions randomized on two different days (Figure 1). One session evaluated wrist *biomechanical properties* (S_*MECH*_) and the second session assessed *wrist position sense* (S_*PERC*_). During both sessions, the right forearm was equipped with bipolar electrodes over the muscle bellies of FCR, FCU, and ECR (Perotto, 2011). Next, the forearm was rested on a support, while the hand grasped the robotic handle. The hand and forearm were secured via VELCRO^®^ straps that restricted forearm movement, isolating the wrist joint and avoiding movement of the hand relative to the handle. On the first experimental day, subjects were encouraged to familiarize with both the device and the experimental conditions for 5 min. Both sessions (S_*MECH*_, S_*PERC*_) were performed entirely with the robotic device and included, in order, a 2-min warm-up, an initial (pre-fatigue) assessment (PRE), a *fatigue task* and four post-fatigue termination assessments (POST 1–4). The warm-up task consisted of moving the robotic handle tracking a target on the screen that follows a Lissajous curve, a commonly used pattern in upper limb tracking exercises (Vergaro et al., 2010), with low-level resistive forces (5N) opposing the motion. After the warm-up and for the remaining duration of the experimental session, subjects were blindfolded both to reduce the cognitive load and to ensure comparable conditions between the two sessions. Each assessment phase (PRE, POST 1–4) involved both a *force assessment* and the evaluation of a specific component related to wrist function. This latter depended on the session (S_*MECH*_ or S_*PERC*_). On the second day of testing, subjects underwent the session they had not yet performed. The assessments were performed before the *fatigue task* (PRE), immediately after (POST 1), and three more times (POST 2–4), with a 30-s rest time between them. To evaluate the rating of perceived fatigue immediately post task termination, subjects rated their perceived level of fatigue on a scale from 0 (“no fatigue”) to 10 (“extreme fatigue”). The entire duration of the protocol, from PRE to POST 4, was less than 20 min.

During each *force assessment*, subjects performed a maximum grip force trial and two maximum voluntary isometric wrist contractions (MVC) in both flexion and extension, using the robot. The force assessment lasted 30 s. During S_*MECH*_, *biomechanical properties* were evaluated through the delivery of position-controlled displacements in the wrist flexion-extension direction and a mechanical impedance model of the wrist joint (Falzarano et al., 2021). These external displacements were characterized by small amplitude and high velocity (100°/s), causing wrist rotation of ±10° from neutral (0°). Throughout this assessment, subjects were instructed to not intervene or resist the perturbation, and to assume a natural grasp. During S_*PERC*_, the *wrist position sense* was tested by an active paradigm. Starting from the neutral position, subjects actively moved the end-effector toward flexion until they haptically perceived the target position as a virtual wall (*criterion movement*). Then, after being returned by the robot to the neutral position, subjects replicated the previously assumed joint configuration, in absence of the target virtual wall (*matching movement*). Each position sense assessment involved 12 targets, whose positions were randomly chosen using a Gaussian distribution centered at 15° (± 1°) from the neutral position. The temporal duration of both assessments (biomechanical properties and position sense) was comparable (2.5 min each).

During the *fatigue task*, which was a modification of Albanese et al. (2021a), subjects performed continuous reaching movements along the FE plane, against a constant resistive torque exerted by the robotic device. This resistive torque opposed flexion movements and facilitated extension movements toward the neutral position. Torque was 60% of each subject’s wrist flexion MVC assessed in PRE. Starting from the neutral wrist position, subjects had to overcome the resistance and reach the target placed at 45° of flexion as fast as possible. Since subjects were blindfolded, targets were perceived through both haptic walls and audible feedback. Each time the target was reached, three sounds could be presented: a “beep,” a “hold,” or a “release” warning. These latter two alerted subjects to modify their grip on the sensorized handle because it fell outside the range of 70–80% of their maximum grip force exerted in PRE. This range of grip was chosen by analyzing data collected in our previous study (Albanese et al., 2021a). Although no grip constraint was given to subjects during that previous study, we detected that, when performing the fatigue task, on average, subjects naturally grasped the handle within that range of force. For the present study, we chose to control for this condition to ensure further repeatability of experimental conditions across subjects and sessions. Specifically, the “hold” feedback occurred when subjects performed the task while grasping the handle with a grip force lower than 70% of the maximum grip force exerted in PRE. This feedback warned them to increase their grip in the following movements. The “release” feedback occurred when the grip was greater than 80% of the maximum grip force exerted in PRE, indicating the subject should reduce the grip. For each completed flexion movement, the sEMG signals of the flexors were band-pass filtered (10–450 Hz) and mean frequency computed. A real-time algorithm was implemented to detect when the mean frequency of either FCR or FCU dropped below 60% of the maximum mean frequency during the fatigue task. When the desired threshold was achieved on two consecutive movements, the robot received a trigger signal and, as soon as subjects were back to the neutral position, the task terminated automatically. The 60% threshold was chosen as the value that ensured all subjects finished the fatigue task and guaranteed a task duration of a few minutes.

### Data Analysis

Joint angular displacements and grip sensor data were filtered with a 6th order Savitzky-Golay filter (10 Hz cut-off frequency), while raw sEMG data were band-pass filtered (10–450 Hz) with a 2nd order Savitzky-Golay filter.

During the *fatigue task*, the root mean square [RMS (mV)] and the mean frequency [MF (Hz)] of the sEMG signals for each flexion movement were measured. Since each subject performed a different number of trials, both outcome measures were resampled to have 100 time points for each subject and these distributions were fitted with a 1st order polynomial function (y = px + c). Concerning fitting parameters, relevant outcomes were *r*^2^, as a measure of goodness of fit, and the slope [p_*MF*_ (mV/sample) or p_*RMS*_ (Hz/sample)], as a metric to compare the rate of change over samples of MF or RMS in different muscles in the same subject. Since we were not interested in comparing raw MF and RMS values, but only their slopes, each subject’s data was not normalized before fitting.

During each *force assessment* (PRE, POST 1–4), the outcome measures were: (1) change in maximum grip force [% of the initial (PRE) maximum grip force]; (2) wrist flexion and extension MVC, measured in Newtons and computed as the ratio of the current delivered by the robotic control unit and the corresponding lever arm of the robotic handle; (3) RMS and MF of the sEMG signals during MVCs.

For the extraction of the *biomechanical properties* of the wrist assessed during PRE and POST 1–4 of S_*MECH*_, we used a linear second-order mass-spring-damper model of the wrist joint that includes the moment of inertia [I (kgm^2^)] and the corresponding viscoelastic properties of the human wrist joint: viscosity [B (Nms/rad)] and stiffness [K (Nm/rad)] parameters (for further and more detailed information refer to the study Falzarano et al., 2021):


(1) $$\tau -\tau_{0}=I\,\left(\ddot{\theta }-{\ddot{\theta }}_{0}\right)+B\,\left(\dot{\theta }-{\dot{\theta }}_{0}\right)+K\,\left(\theta -\theta_{0}\right)$$

τ (Nm) is the torque exerted by the robot to the hand for the FE displacements (ensuring that hand placement, and therefore lever arm, was kept similar among subjects) estimated by measuring the current delivered by the control unit to the actuators, with the current signal smoothed by a 1st order Savitzky-Golay filter (∼20 Hz cut-off frequency); τ_0_ is the torque maintained before starting the displacement; θ (rad) is the angular displacement measured by the FE motor encoder; the recorded angular displacements were filtered by a 3rd order Savitzky-Golay filter (∼12 Hz cut-off frequency) and, from its differentiation, we obtained the velocity $\dot{\theta }$ (rad/s) and acceleration θ (rad/s^2^); ${\dot{\theta }}_{0}$ and θ_*0*_ are the initial angular velocity and acceleration, respectively, and both have null values in the adopted experimental protocol. For each position-controlled displacement, the “best fit” parameters (B, K) of the mechanical impedance were estimated using a least square approximation procedure capable of minimizing the mean square error between the torques measured experimentally and those predicted by the model. We excluded from the analysis the disturbances whose torque signal differed statistically from the others of the same type (flexion or extension movements). For each subject, B and K were averaged for each type of movement (flexion and extension). Moreover, the reflex responses (within 100 ms from the onset of the displacement) of the stretched muscles were analyzed, i.e., the stretch reflex responses of the flexors were analyzed for the position-controlled movements in extension and vice versa for the extensor muscle. In particular, sEMG signals were band-pass filtered (25–250 Hz, two-pass) with a 2th order Savitzky-Golay filter, full-wave rectified, and normalized to the mean activity during 200 ms before the onset of perturbation (Pruszynski et al., 2008). Additionally, we temporally shifted the sEMG signals by a fixed delay of 10 ms, which was the time from delivery of the perturbation to the onset of hand acceleration. We were interested in measuring average muscle activity in the recorded muscles during five temporal epochs from the onset of each displacement: (1) baseline, BASE (<100 ms); (2) short-latency reflex, R1 (20–45 ms); (3) mid-latency reflex, R2 (45–75 ms); (4) long-latency reflex, R3 (75–105 ms); (5) voluntary response, VOL (120–180 ms) (Pruszynski et al., 2008).

During PRE and POST 1–4 of S_*PERC*_, *wrist position sense* was evaluated in terms of Matching Error [ME (°)] and Error Bias [EB (°)]. ME was the absolute difference between target and matching position. EB was the signed difference, with positive and negative values indicating over- and undershot movements, respectively. Since the task was completely active, additional measures were computed to investigate other unconstrained factors that could have influenced perception. During the matching phase, peak velocity [v_*peak*_ (°/s)] and grip as a percentage of the initial (PRE) maximum grip force [grip_*M*_ (%)] were calculated. Since we did not know *a priori* the dynamics of fatigue-related changes in position sense, we hypothesized that each post-fatigue assessment could have exceeded the ideal task duration, thus flattening outcome changes among subsequent assessments. For this reason, we repeated the analysis twice, first considering outcome measures in all 12 trials performed in each assessment, and then including only the first six trials (early trials).

### Statistical Analysis

Shapiro-Wilk Tests verified the normality of the data measured during each force assessment. Two-way Repeated Measures ANOVAs evaluated differences in the measurements (grip, MVC, RMS, MF) across assessments (PRE and POST1–4) and sessions (S_*MECH*_, S_*PERC*_). Two-way Repeated Measures ANOVAs compared slopes of fitting functions (p_*RMS*_, p_*MF*_) computed for each muscle (FCR, FCU, ECR) and session (S_*MECH*_, S_*PERC*_) during the fatigue task. In the presence of statistical significance (*p* < 0.05), *post-hoc* pairwise comparisons corrected with Tukey were used to investigate the main effects and interactions. To test the influence of fatigue and displacement direction on the biomechanical parameters (K and B), a Mixed-Effects Model with K (and B) as the dependent variable, *direction* and index of *assessments* (PRE and POST1–4) as independent variables, and subject as the random factor was conducted. Furthermore, for each sEMG signal, a Mixed-Effects Model was used to test the influence of fatigue and how muscle activity changed across the temporal epochs. In this case, the individual muscle activity was the dependent variable, the index of *assessment* (PRE and POST1–4) and the *temporal epochs* (BASE, R1, R2, R3, VOL) were independent variables, and subject was the random factor. To test the influence of fatigue on wrist position sense, a Mixed-Effects Model with the outcome measure (ME, EB, v_*peak*,_ grip_*M*_) as the dependent variable, the index of *assessments* (PRE and POST1–4) as independent variable, and subject as the random factor was conducted. The same analysis was computed including only early trials (6 out of 12) of each position sense assessment. Statistical analysis was performed using the Jamovi Statistical Data Analysis software (JSDA, version 1.6.23).

## Results

### Fatigue Task

**FIGURE 2:**
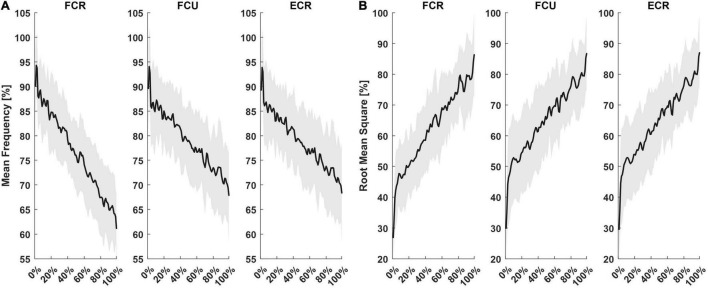
Mean and standard deviation of sEMG outcomes from the three muscles recorded. For each subject, MF **(A)** and RMS **(B)** computed for each flexion movement were resampled on 100 points and presented as a percentage of their maximum.

The fatigue task lasted 78 ± 26 s, for a total of 63 ± 20 flexion movements performed until the algorithm detected the fatigue threshold and terminated automatically. Subjects’ mean rate of perceived fatigue was 8 ± 0.7 out of 10. Figure 2 shows mean MF (Figure 2A) and RMS (Figure 2B) as a percentage of maximum, computed during each flexion movement, resampled to 100 points. Fitting the resampled MF data of each subject with a 1st order polynomial function, the average *r*^2^ values were 0.70, 0.52, and 0.50 for FCR, FCU, and ECR, respectively. Slopes of fitting functions (1st order polynomial functions) helped determine which muscle presented the fastest rate of fatigue. The average slope for MF fittings were, p_*MF*_ = −0.26 Hz/sample for FCR, p_*MF*_ = −0.21 Hz/sample for FCU and p_*MF*_ = −0.20 Hz/sample for ECR. There was a significant difference among muscles (*F* = 7.80, *p* = 0.002), where the decrease in MF was faster for FCR (FCR vs. FCU: *T* = −2.92, *p* = 0.030; FCR vs. ECR: *T* = −2.86, *p* = 0.034) than all other muscles. Concerning RMS, *r*^2^ was 0.60 for FCR, 0.60 for FCU and 0.61 for ECR. RMS slopes were p_*RMS*_ = 1.6*10^–3^ mV/sample for FCR, p_*RMS*_ = 1.3*10^–3^ mV/sample for FCU and p_*RMS*_ = 1.2*10^–3^ mV/sample for ECR. Both p_*MF*_ and p_*RMS*_ showed no statistically significant difference between sessions (S_*MECH*_, S_*PERC*_) or interaction between sessions and muscles (FCR, FCU, ECR).

### Force Assessment

There were no significant differences in maximal isometric wrist extension force across the POST evaluations. However, there was a decrease in maximum grip force (*F* = 6.23, *p* < 0.001) and maximum isometric wrist flexion force (*F* = 3.67, *p* = 0.010). Grip force decreased from POST1 to POST4 (*T* = 3.51, *p* = 0.039), while isometric flexion force significantly decreased from PRE to POST2 and then recovered back to PRE (*T* = 3.19, *p* = 0.046). Both flexor muscles presented a significant decrease in MF immediately after the end of the fatigue task (FCR: *F* = 58.74, *p* < 0.001, POST1 vs. each assessment *p* < 0.001; FCU: *F* = 23.84, *p* < 0.001, POST1 vs. each assessment *p* < 0.001), and a concurrent slight increase in RMS, followed by a decrease in the assessments subsequent to POST1 (FCR: *F* = 5.56, *p* < 0.001, POST1 vs. POST2 *T* = 4.08, *p* = 0.009, POST1 vs. POST3 *T* = 3.70, *p* = 0.019; FCU: *F* = 5.61, *p* < 0.001, POST1 vs. POST4 *T* = 3.56, *p* = 0.024). Interestingly, despite no significant decrease in isometric extension force, the extensor muscle showed the same trend: a significant decrease in MF immediately after the end of the fatigue task (*F* = 28.06, *p* < 0.001, POST1 vs. each assessment *p* < 0.001) and a slight increase in RMS at POST1, with a decrease until POST4 (*T* = 5.21, *p* < 0.001, POST1 vs. POST4: *T* = 3.30, *p* = 0.039). All muscles presented no specific trend of frequency and amplitude change in assessments performed in extension.

### Biomechanical Properties of the Wrist Joint

**FIGURE 3:**
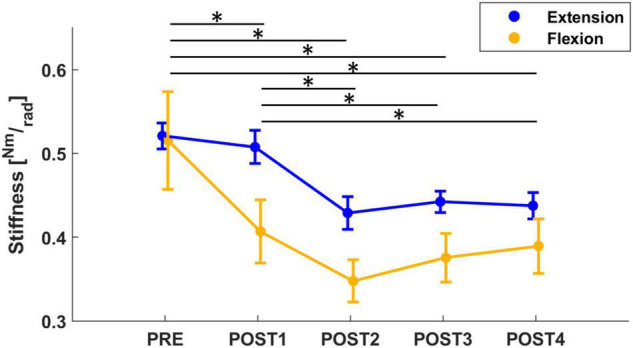
The mean and standard error of wrist stiffness, calculated in both flexion (yellow) and extension (blue) directions. Stiffness significantly decreased from PRE to all post-fatigue assessment sessions, showing a more significant decrease in flexion than extension direction. Significant results (*p* < 0.05), after *post-hoc* analysis, were marked with “*.”

The mixed model of the stiffness values revealed a significant effect of both *assessment* (*F* = 19.20, *p* < 0.001) and *direction* (*F* = 16.62, *p* < 0.001). Corrected *post-hoc* analysis showed that stiffness decreased significantly after fatigue (PRE to POST1 with a decrease of 11%: *T* = 3.21, *p* = 0.013; PRE to POST2 with a decrease of 26%: *T* = 7.12, *p* < 0.001; PRE to POST3 with a decrease of 25%: *T* = 7.0, *p* < 0.001; PRE to POST4 with a decrease of 22%: *T* = 6.01, *p* < 0.001; POST1 vs. POST2: *T* = 3.99, *p* < 0.001; POST1 vs. POST3: *T* = 3.84, *p* = 0.001; POST1 vs. POST4: *T* = 2.88, *p* = 0.04) and did not return to pre-fatigue at the end of the experiment (approximately 12 min after the fatigue task). Furthermore, a corrected *post-hoc* analysis showed that stiffness measured in the flexion direction decreased significantly more than in extension (*T* = 4.08, *p* < 0.001), both starting from pre-fatigue values that were not significantly different between directions. In both directions, the lowest values were reached during the POST2 assessment (approximately 4 min after fatigue, Figure 3). In flexion, stiffness reduced from 0.516 Nm/rad in PRE to 0.369 Nm/rad (−26%) and in extension, stiffness reduced from 0.562 Nm/rad in PRE to 0.429 Nm/rad (−21%).

**FIGURE 4:**
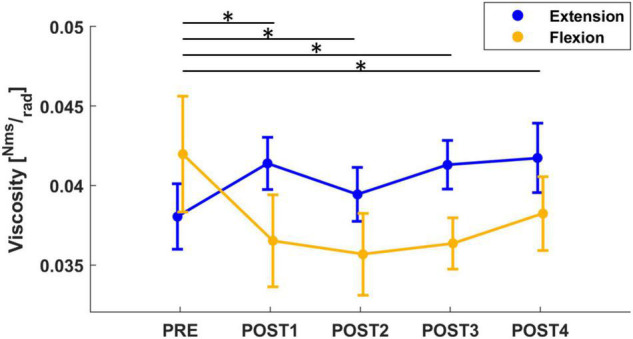
The mean and standard error of wrist viscosity, calculated in both flexion (yellow) and extension (blue) directions. Viscosity computed in flexion significantly decreased from PRE to all post-fatigue assessment sessions (the significant results after *post-hoc* analysis, marked with “*,” refer to the flexion viscosity: yellow line), whereas viscosity in extension showed an increasing tendency after fatigue.

Concerning the viscosity parameter, the mixed model results revealed a significant effect of both *assessment* (*F* = 4.67, *p* < 0.001), *direction* (*F* = 20.12, *p* < 0.001) and an *interaction* between these 2 outcomes (*F* = 5.70, *p* < 0.001). A corrected *post-hoc* analysis confirmed the effect of assessment sessions on the viscosity parameter (PRE vs. POST1: *T* = 3.12, *p* = 0.019; PRE vs. POST2: *T* = 3.71, *p* = 002; PRE vs. POST4: *T* = 3.46, *p* = 0.006). Immediately after the fatigue task, a slight decrease in viscosity was detected, which then returned to pre-fatigue at the end of the experiment (approximately 12 min after the fatigue task). In addition, we further investigated the effect of direction on viscosity. Pre-fatigue viscosity did not change with direction; whereas, after the fatigue task, the behavior of viscosity changed with direction (*T* = 4.49, *p* < 0.001): viscosity measured in flexion significantly decreased after fatigue (PRE to POST1 with a decrease of 23%: *T* = 5.22, *p* < 0.001; PRE to POST2 with a decrease of 26%: *T* = 5.33, *p* < 0.001; PRE to POST3 with a decrease of 22%: *T* = 4.98, *p* < 0.001; PRE to POST4 with a decrease of 22%: *T* = 4.98, *p* < 0.001) whereas viscosity measured in extension showed an increasing (not significant) tendency immediately after fatigue (Figure 4). In addition, the mean grip force decreased across the different assessment sessions (*F* = 44.7, *p* < 0.001). In particular, grip force significantly decreased from 29.4% MVC in PRE to 25.8% MVC, 20.6% MVC and 18% MVC in POST1 (*T* = 5.00, *p* < 0.001), POST2 (*T* = 9.73, *p* < 0.001) and POST3 (*T* = 11.3, *p* < 0.001), respectively, and remained at 18% MVC in POST4.

**FIGURE 5:**
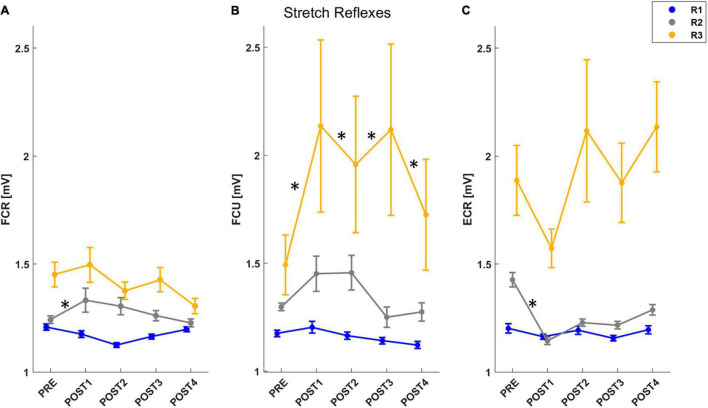
The mean and standard error of muscle activity for each muscle stretched by the perturbation: FCR **(A)**, FCU **(B)**, ECR **(C)**. For each figure, muscle activity is reported for R1 (short-latency reflex): 20–45 ms (blue line), R2 (mid-latency reflex): 45–75 ms (gray line), R3 (long-latency reflex): 75–105 ms (yellow line). After *post-hoc* analysis, significant differences (*p* < 0.05) in muscle activity for each temporal epoch compared with PRE assessment were marked with “*.”

To assess the stretch reflex time periods for the recorded muscles, we analyzed each muscle individually (Figure 5). For FCR, the mixed model results for the muscle activity revealed a significant effect of both *assessment* (*F* = 2.81, *p* = 0.024), *temporal epochs* (*F* = 100.64, *p* < 0.001) and an *interaction* between these two outcomes (*F* = 1.85, *p* = 0.035). A *post-hoc* analysis confirmed that there was an increase in muscle activity immediately after fatigue (PRE vs. POST1: *T* = 3.08, *p* = 0.002), and, muscle activity in the R2 window increased significantly until 4 min after the task (PRE vs. POST1: *T* = 3.25, *p* = 0.001; PRE vs. POST2: *T* = 2.20, *p* = 0.028) (Figure 5A). For FCU, the mixed model results for the muscle activity revealed a significant effect of both *assessment* (*F* = 2.75, *p* = 0.027), *temporal epochs* (*F* = 74.39, *p* < 0.001) and an *interaction* between these two outcomes (*F* = 2.45, *p* = 0.003). A *post-hoc* analysis confirmed that there was an increase in muscle activity immediately after fatigue until 8 min (PRE vs. POST1: *T* = 2.77, *p* = 0.006; PRE vs. POST2: *T* = 2.07, *p* = 0.039; PRE vs. POST3: *T* = 2.95, *p* = 0.003), and, muscle activity in the R3 window increased significantly after the task until the end of the experiment (PRE-POST1: *T* = 2.55, *p* = 0.011; PRE-POST3: *T* = 4.23, *p* < 0.001; PRE-POST4: *T* = 2.37, *p* = 0.018) (Figure 5B). For ECR, the mixed model results for the muscle activity revealed a significant effect of both *assessment* (*F* = 12.73, *p* < 0.001), *temporal epochs* (*F* = 203.26, *p* < 0.001) and an *interaction* between these two outcomes (*F* = 8.57, *p* < 0.001). A *post-hoc* analysis confirmed that there was a decrease in muscle activity immediately after fatigue (PRE vs. POST1: *T* = 3.18, *p* = 0.001), and, muscle activity in the R2 window decreased significantly immediately after the task (PRE-POST1: *T* = 1.86, *p* = 0.046) (Figure 5C).

### Wrist Position Sense

**FIGURE 6:**
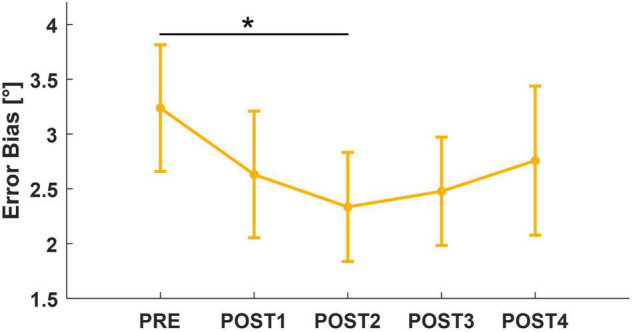
Mean and standard error for Error Bias, computed considering the first six trials of each assessment. After *post-hoc* analysis, significant results (*p* < 0.05) were marked with “*.”

Limiting the analysis to the pairwise comparison between PRE and each assessment following the fatigue task, we found a significant decrease in both EB and ME between PRE and POST2 (EB: *T* = 2.12, *p* = 0.034; ME: *T* = 2.51, *p* = 0.013). Interestingly, EB and ME led to similar results because of subjects’ tendency to overshoot the target while matching. However, there was no significant effect of *assessment* (ME: *F* = 2.23, *p* = 0.064; EB: *F* = 2.14, *p* = 0.074) for both metrics. When considering trial number (1–12) as a factor in the mixed model, both outcomes revealed an effect of trial (ME: *F* = 2.13, *p* = 0.016; EB: *F* = 2.65, *p* = 0.002). For this reason, we repeated the mixed model analysis considering only the first six out of 12 trials of each assessment session. This revealed a significant effect of *assessment* for EB (*F* = 2.48, *p* = 0.043). Corrected *post-hoc* analysis showed that accuracy increased (EB decreased toward zero) after the fatigue task, reaching its peak around 4 min after the end of the task (PRE vs. POST2: *T* = 2.89, *p* = 0.041). At POST3, position sense returned comparable to PRE (Figure 6). In addition, to look for the presence of changes in the strategy used to accomplish the task when performing the *matching movement*, the peak of velocity and the grip force exerted on the handle decreased, respectively, from 46.1 to 40.5°/s and from 21.7 to 19.1% of maximum grip. Both metrics reached their minimum in POST2, i.e., 4 min after the end of the task (v_*peak*_: *F* = 5.70, *p* < 0.001; *post-hoc*: PRE vs. POST2: *T* = 4.60, *p* < 0.001; PRE vs. POST3: *T* = 3.20, *p* = 0.013; PRE vs. POST4: *T* = 2.94, *p* = 0.028; grip_*M*_: *F* = 6.73, *p* < 0.001; *post-hoc*: PRE vs. POST2: *T* = 4.20, *p* < 0.001; PRE vs. POST3: *T* = 3.20, *p* = 0.008; PRE vs. POST4: *T* = 4.32, *p* < 0.001).

## Discussion

The purpose of this work was to evaluate the effects of forearm muscle fatigue on wrist flexion/extension MVC, biomechanical properties of the wrist joint, and wrist position sense. These measures were evaluated quantitatively using a robotic device before and at four time-points after a fatigue task implemented using the same robot. Notably, the employment of the robotic device during the entire protocol was able to assure accuracy and repeatability in timings, postures, and, consequently, outcome measures (Albanese et al., 2021a). The fatigue task consisted of a short-term submaximal dynamic wrist flexion/extension task where the wrist robot generated a torque opposing flexion movements. As hypothesized, the fatigue task led to a decrease in flexion MVC, occurring 4 min after the end of the task. At that same time, wrist stiffness decreased and position sense improved over the same time period. In the following sections, we discuss the main findings of each experimental phase and the mutual relationship between fatigue-related changes in the different components assessed.

### Fatigue Task

Throughout the experiment, muscle activity was recorded from three forearm muscles (two flexors: FCR, FCU, and one extensor: ECR). A novelty of the protocol was that the fatigue threshold was not based on perceived fatigability, i.e., the subjective level of fatigue reported by subjects, but relied on an objective measurement associated with the state of fatigue. In our work, this was defined when the MF of either flexor muscle fell below a fixed threshold. During sustained muscle contractions, the production of lactate increases, thus leading to intra- and extra-cellular pH changes (Fitts and Holloszy, 1976). Indeed, ion concentrations influence membrane excitability of the muscle tissue and, consequently, the propagation of an action potential over the muscle fiber, evaluated as muscle fiber conduction velocity (MFCV). When experiencing fatigue, reduction in MFCV is responsible for a shift in sEMG spectral density function toward lower frequency components (Mortimer et al., 1970; Merletti et al., 1990). During fatiguing tasks, muscle fatigue is characterized not only by the two features mentioned above (decrease of MFCV and mean frequency of the sEMG), but also by an increase in the amplitude of the sEMG signal (Cifrek et al., 2009). However, sEMG amplitude has been observed to increase during sub-maximal efforts, but decrease during maximal efforts (Carr et al., 2016). Given this, we chose to terminate the proposed fatigue task only on the basis of a specific percentage decrease in muscle mean frequency.

To verify how the three muscles were actually targeted by fatigue, we studied their behavior in terms of MF and RMS amplitude throughout the fatigue task. Consistently with literature on the identification of the fatigue state (Luttmann et al., 2000), we found a decrease in MF and an increase in RMS amplitude in all three muscles from the beginning to the end of the task. FCR was the most affected flexor muscle, presenting a faster rate of change than FCU. It is known that resistance to fatigue is significantly influenced by muscle composition. Muscles composed of slow-twitch (ST) fibers possess a greater resistance to fatigue than those containing primarily fast-twitch (FT) (Edström and Kugelberg, 1968; Kugelberg and Edstrom, 1968; Baldwin and Tipton, 1972). Because of a paucity of studies about fiber composition in human wrist muscles (McIntosh et al., 1985; Mizuno et al., 1985), we can only speculate that our results may be due to a greater quantity of FT fibers in FCR than in FCU. Another possible explanation could be related to differences in muscle lines of action. As described by Bawa et al. (2000), the pulling direction of FCR is closer to pure flexion than FCU, which could partly explain a greater involvement of FCR in the task. Interestingly, the fatigue task led to fatigue of both flexors and extensors, although the two muscle contractions were different due to the direction of applied torque. Muscle work was required to oppose the resistive torque in flexion; this contraction was concentric for the flexors and eccentric for the extensors. Both contractions led to changes in MF and RMS, but greater changes were found in muscles fatigued under concentric contractions, which confirms previous results for upper limb concentric and eccentric exercise (Linnamo et al., 2000). Additionally, although the fatigue task was designed to evoke fatigue in the flexors, the extensors also act as joint stabilizers and would be active during flexion (and grasping). This could partly explain why, in our work, ECR exhibited signs of fatigue. Literature has demonstrated that extensor involvement particularly increases in dual tasks, such as performing a flexion movement while grasping a handle (Forman et al., 2019).

### Force Assessment

We found a decrease in isometric wrist flexion force at approximately 4 min after the fatigue task, while isometric wrist extension force did not change during post fatigue assessments. It has been demonstrated that ion concentration alterations, particularly peaks in blood lactate concentration (Tesch et al., 1978), cause impaired function of the contractile proteins needed to generate muscle forces (Allen et al., 2008; Fitts, 2016). Indeed, previous works (Tesch and Wright, 1983; Tanimoto and Ishii, 2006) have found that, after both maximal (Tesch and Wright, 1983) and submaximal resistance exercise (Tanimoto and Ishii, 2006), the greatest peak in blood lactate concentration is about 3–4 min after fatigue, thus explaining why force changes were not found immediately after the task. Additionally, lower peaks of lactate found after eccentric exercises (Linnamo et al., 2000) could partially explain why the performed task did not demonstrate a decrease in isometric extension force. Concerning the recorded muscle activity, we reported a decrease in MF and an increase in RMS immediately after fatigue, both of which returned to baseline within 4 min after the end of the task. The reason for the sEMG amplitude increase may be related to an enhancement of both motor unit recruitment and changes in discharge rates to compensate for the decline in muscle fiber force generation (Yoon et al., 2013). The decrement in MF is partially related to those ion alterations that impair contractile function and lead to the decrease in isometric force output. However, changes in the sEMG signal were evident earlier than the force changes (POST1 vs. POST2). Indeed, the biochemical ion distribution also determines alterations in membrane excitability and, consequently, in the velocity of action potential conduction. Although conduction velocity is a factor that affects the shift toward lower frequencies (i.e., MF decrease), previous works have found that median frequency decrease exceeds the decrease in conduction velocity (Krogh-Lund and Jørgensen, 1992, 1993). In addition, motor unit (MU) synchronization (Kleine et al., 2001; Boonstra et al., 2008) is another factor that could have contributed to the shift toward lower frequencies.

### Biomechanical Properties of the Wrist Joint

Recent research suggests that assessing modest changes in muscle stiffness may help detect the early stages of muscle fatigue (Andonian et al., 2016; Siracusa et al., 2019), damage (Lacourpaille et al., 2014), or disease (Rasool et al., 2018). Indeed, identifying patterns of muscle fatigue related to changes in muscle stiffness could aid in the diagnosis of muscle conditions (e.g., neuromuscular disorders) or provide insights for muscle rehabilitation. Furthermore, since it is unknown if changes in muscle biomechanical properties aid in restoring force transmission capacities during the recovery period, we examined the fatigue dependence to understand better discrepancies in muscle stiffness changes after fatigue and over the recovery period.

Our work demonstrated that the fatigue state can affect biomechanical properties of the wrist joint. Specifically, stiffness, computed in both directions, significantly decreased after fatigue. This result is consistent with the literature, with two possible explanations suggested by muscle physiology: (1) the inability of sarcomere cross-bridges to generate the same amount of net force as they did before fatigue and (2) a fatigue-related change in intramuscular temperature (Lakie and Robson, 1988; Chalchat et al., 2020; Mallette et al., 2021). However, stiffness calculated in the flexion direction decreased significantly more than extension and this difference is probably justified by the direction of applied torque during the fatigue protocol. For both directions, the lowest stiffness was reached approximately 4 min post-fatigue, which corresponded with the largest decrease in isometric wrist flexion MVC from pre-fatigue (Qita and Kearney, 2000). Interestingly, stiffness did not recover to the pre-fatigue value at the end of the experiment, which lasted approximately 12 min after the end of the fatigue task. This finding is supported by other work results that showed the thixotropic muscle properties affected by fatigue return to control levels approximately within 60–90 min (Lakie and Robson, 1988). Moreover, because a robust negative correlation between grip force and wrist stiffness has been demonstrated (Falzarano et al., 2021), we also investigated changes in the natural grip force that subjects exerted on the robotic handle during assessments. We found a significant decrease in grip force over the assessments of wrist biomechanical properties.

Viscosity exhibited different behaviors for each direction. In the pre-fatigue state, viscosity computed in flexion and extension did not differ, whereas, after fatigue, there was a decreasing trend of viscosity measured in the flexion direction and an increasing trend of viscosity measured in the extension direction. We did not find many works investigating the viscosity after fatigue and no work differentiating viscosity based on directions. Some researchers (Cornu et al., 1997; Zhang and Zev Rymer, 2001; Chalchat et al., 2020) showed that, after a fatigue task, viscosity increased, which may be related to a reduction in muscle fiber relaxation and a lower rate of cross-bridge detachment, thereby affecting muscle function. One possible explanation for why only viscosity measured in extension increased after fatigue could be that, for our specific fatigue task, the extensors were fatigued eccentrically while the flexors were fatigued concentrically. As reported in the literature, fatigue during eccentric contractions causes more profound changes in some aspects of muscle function than concentric contractions (Newham et al., 1983). Indeed, eccentric contractions have been shown to have significant effects initially on the contractile properties and force-generating abilities of muscles, and result in delayed-onset pain. This occurs because high forces are generated by a few muscle fibers, and the transmission of these forces to non-contractile tissues causes subsequent mechanical damage. Differently, a decrease in viscosity, as occurred in the flexor muscles after fatigue, may occur as a result of movement, inducing a reorganization of the more mobile constituents of muscle tissue, such as polysaccharides and water (Mcnair et al., 2001).

This work also evaluated changes in muscle activity and stretch reflexes during the assessment of the biomechanical properties of the wrist joint. We could study stretch reflexes since the velocity of the perturbations used for the estimation of the biomechanical properties was 100°/s, and it was sufficient to elicit responses (Hufschmidt and Mauritz, 1985). Therefore, we analyzed the stretch reflexes of the muscles stretched by the angular perturbation. We evaluated stretch reflexes in the flexor muscles when the displacement was directed in the extension direction and vice versa for those in the extensor muscle. We found changes in the amplitude of the sEMG activity after fatigue. Specifically, the amplitude of the short-latency reflex (SLR) did not change in any of the muscles. The mid-latency reflex (MLR) increased immediately after the fatigue task in FCR (concentrically fatigued) and decreased in ECR (eccentrically fatigued). As with viscosity, this result may be accounted for by how muscles were fatigued in this protocol; in particular, eccentrically fatigued muscles may undergo morphological changes in the intracellular proteins with a subsequent attenuation of force-production due to impaired cross-bridge binding, leading to a reduction in muscle activity (Kuitunen et al., 2002). Both these changes rapidly recovered, with a return to control (pre-fatigue) activity within 4 min, in agreement with the findings of Balestra et al. (1992). Finally, the long-latency reflex (LLR) increased immediately after the fatigue task in FCU, which was also concentrically fatigued, and remained constant until the end of the experiment. Since after the fatigue task there was an increase in sEMG activity in both flexor muscles during both the R2 and the R3 temporal epochs, it could be concluded that our task involved multiple mechanisms. Indeed, whereas muscle activity during SLR (R1) is associated entirely with spinal circuits, muscle activity during both MLR (R2) and LLR (R3) depends on contributions from supraspinal structures such as the primary motor cortex (Kurtzer et al., 2010). However, a previous study investigating the different components of stretch reflexes following voluntary fatigue and electrically induced fatigue showed that the MLR and LLR reflex components had two different behaviors: MLR decreased during voluntary contraction without significant changes in LLR, whereas in electrically induced fatigue, LLR improved without significant changes in MLR (Balestra et al., 1992). These results imply that the reflex MLR and LLR components are distinct entities in the long-latency complex. The latter point is also confirmed, in our study, by the fact that MLR returns to control values in about 4 min, whereas LLR remained above control at about 12 min after the fatigue protocol. Therefore, we can conclude that the contractions required by the fatigue task involve fatigue of the reflex components as well and, additionally, that the long-latency reflex components are composed of at least two entities mediated by different peripheral afferents.

### Wrist Position Sense

We found significantly reduced errors in matching target positions 4 min after the end of the fatigue task (POST2). This increase in accuracy is in agreement with (Mugnosso et al., 2019), whose robotic testing protocol aimed at fatiguing wrist flexor muscles and resulted in post-fatigue matching positions closer to the target in flexion. The same trend, and recovery to pre-fatigue values, was found when analyzing only the first six trials performed during the position matching task, particularly for Error Bias. The reason why the effect was more noticeable in the early trials (1–6) of each assessment, may be due to the ratio between the position matching task duration and the temporal distance between the following assessments. We may hypothesize that, during the time necessary to accomplish the task (2.5 min), an adaptation process was initiated that led to recovery wrist position sense along the direction fatigued concentrically. Since the timing and the temporal duration of this process was *a priori* unknown to us, and given that some additional cognitive fatigue could have contributed while performing the task blindfolded (Abd-Elfattah et al., 2015), 6 trials of position matching were likely closer to the appropriate task duration than the proposed 12 trials.

Muscle spindles are the main receptors involved in position sense tasks (Proske and Gandevia, 2012) and are the only proprioceptors whose output can be modified from the central nervous system (Ashton-Miller et al., 2001). In the literature, there is evidence that increased firing patterns of alpha and gamma motoneuron lead to an increase in proprioceptive sensitivity related to muscle spindles (Jami et al., 1980). Other mechanoreceptors, located in skin and ligaments, are not modulated by central commands, but sensitivities can still be influenced by other factors. Among them, vasodilation derived from increased body temperature might improve sensitivity (Bartlett and Warren, 2002) with high-speed flexion movements that might act as a warm-up exercise, improving overall proprioception (Ju et al., 2010). Apart from changes in receptor sensitivity, other factors that could influence the results are related to movement and posture variability. Given that the position matching task did not involve passive motion in flexion, active motion speed could have had an influence. Since, after fatigue, the peak velocity decreased when matching, subjects might have perceived to move longer in space when moving for longer times, thus resulting in spatially shorter movements (Goble, 2010). The influence of this component could be avoided by using a different task, like a passive joint position matching (Lönn et al., 2000). However, the choice to rely on active position matching was motivated by the core idea of selecting a task close to real activities of daily living or work actions, during which there is the need of replicating the same active movement over time. Finally, another source of variability could be contributed to how subjects naturally grasped the handle. Since the muscles involved in the fatigue task also contribute to grasping, the natural grip was likely influenced by the performed fatigue task, thus leading the post-task natural grip to be more relaxed when fatigued. However, a study found that movement accuracy was higher while exerting lower grip forces (Finneran and O’Sullivan, 2013), thus grip could certainly have been a possible source of post-fatigue changes in perception.

### Biomechanical Properties and Position Sense Reciprocal Influence

This work is the first study to describe a repeatable protocol to study how the same fatigue task affected different components of wrist functionality, all assessed at the same time points. For this reason, it is worth discussing how results found in biomechanical properties and position sense might be related to each other. Firstly, the decrease in joint stiffness found after fatigue may also be associated with the improvements detected in the position sense. Indeed, as suggested by Ju et al. (2011), less stiffness implies less interference when actively repositioning the joint during the matching phase. Additionally, we found an increased stretch reflex detected in flexor muscles after the fatigue task (mid-latency for FCR and long-latency for FCU). The increased stretch reflex of concentrically fatigued muscles involves Ia afferents fibers in the posterior nerve root, which directly or indirectly increase the firing of alpha motoneurons (Smyth and Peacock, 2003). As pointed out when discussing results obtained in the position sense assessments, muscle spindles output can be modified centrally. Particularly, in accordance with our results, it has been demonstrated that, when alpha and gamma motoneuron firing increases, muscle spindles enhance their sensitivity and consequently position sense accuracy (Jami et al., 1980; Durbaba et al., 2003).

## Conclusion

Overall, our results confirmed previous understanding of the effects of fatigue on biomechanical properties and position sense, and integrated new findings on the temporal dynamics of these transient changes. Although a large body of literature focuses on the effects of fatigue, the protocols performed are largely disparate, thus leading to the impossibility of assessing the effects of fatigue on different aspects of upper limb function. For these reasons, we believe that this method can be considered the first step toward a more accurate characterization of the effects of forearm fatigue on wrist function. Exploiting robotics, we implemented a repeatable protocol, which could be tailored to a widely variable population. This work represents a preliminary validation on a uniform population of healthy subjects as a baseline for future applications on wider samples of subjects, such as workers, athletes or persons suffering from neurological disorders. Although we limited our analysis to some specific aspects of wrist function, a future perspective may be to broaden the set of measures assessed, considering other aspects related to motor performance or testing the joint along degrees of freedom other than those involved in the present task.

## Data Availability Statement

The raw data supporting the conclusions of this article will be made available by the authors, without undue reservation.

## Ethics Statement

The studies involving human participants were reviewed and approved by the Liguria Region (n.222REG2015). The patients/participants provided their written informed consent to participate in this study.

## Author Contributions

GA, VF, and JZ designed the study and formulated the experimental question. GA and VF collected the data, performed the data analysis and statistics, and wrote the manuscript. JZ supervised the study. MH, PM, and JZ participated in results interpretation and manuscript revision. All authors approved the final version of the manuscript.

## Conflict of Interest

The authors declare that the research was conducted in the absence of any commercial or financial relationships that could be construed as a potential conflict of interest.

## Publisher’s Note

All claims expressed in this article are solely those of the authors and do not necessarily represent those of their affiliated organizations, or those of the publisher, the editors and the reviewers. Any product that may be evaluated in this article, or claim that may be made by its manufacturer, is not guaranteed or endorsed by the publisher.
